# Do They Feel Heard? A Study of Canadian Secondary School Students’ Voices Around Violent Extremism

**DOI:** 10.1177/00220574251318312

**Published:** 2025-02-03

**Authors:** Maihemuti Dil Dilimulati, Helal Hossain Dhali, Ratna Ghosh

**Affiliations:** 1 5618 Concordia University, Montreal, QC, Canada; 2 McGill University, Canada; 3 Université du Québec à Montréal (UQAM), Canada

**Keywords:** preventing/countering violent extremism (P/CVE), Canadian secondary school students, ethics of care, moral disengagement, the role of education, Islamophobia

## Abstract

This paper examines the perspectives and experiences of Canadian secondary school students on violent extremism. Through interviews with students (*n* = 36), the study highlights a significant lack of critical understanding of the topic. Although students perceive their teachers as competent, discussions on violent extremism are notably absent in classrooms, leading students to over-rely on informal sources such as social media, often rife with misinformation. Furthermore, many students tend to overemphasize Islamist extremism while showing no knowledge about right-wing or other forms of extremism. Some Muslim students report facing identity crises and discrimination due to prevailing discourses around Islam and violence.

## Introduction

Recently, the global landscape has witnessed an alarming rise in incidents of violent extremism, posing significant challenges to peace, security, and social cohesion in various contexts. In 2016, Public Safety Canada (2017) had already highlighted a concerning trend in Canadian security, noting that the number of Canadians involved with foreign extremist groups in the Middle East had reached 190. The Canadian Incident Database reports that between 2003 and 2016, right-wing extremists perpetrated an average of 3.3 violent incidents annually, with a notable escalation in frequency over the same period (Parent & Ellis, 2016). The proliferation of such ideologies is underscored by estimates suggesting the presence of at least 130 active right-wing extremist groups across Canada around 2015 (Perry & Scrivens, 2016).

The notable increase in hostility and violent incidents targeting minority religious groups, including Muslims and Jews, during the first Trump administration in North America is widely regarded as not coincidental. Some scholars have coined the term “Trump effect” to describe this trend (Perry & Scrivens, 2019, p. 143). In Canada, for example, between 2019 and 2020 alone, the number of police-reported hate crimes motivated by right-wing ideologies surged by a staggering 80%, rising from 884 to 1594 incidents (Wang & Moreau, 2022). Horrific events such as the Quebec Mosque attack in 2017 and the London, Ontario truck attack in 2021, resulting in the tragic loss of six and four innocent lives, respectively, serve as prominent examples of this phenomenon (CBC, 2022).

A 2020 Institute for Strategic Dialogue report revealed the widespread presence of right-wing extremist channels, pages, groups, and accounts across seven social media platforms in Canada, surpassing 6600 in number (Davey et al., 2020). Recent years have witnessed a further rise of far-right extremism within Canada, with an increased youth involvement in right-wing extremist movements (Chen, 2024; Government of Canada, 2022), including post-secondary campuses (Haye & Patrick, 2023). Some scholars have noted that right-wing extremists have perpetrated a more significant number of violent attacks in Canada and the United States than any other form of extremism since 9/11 (Bjorgo & Jacob, 2019; Budning, 2023). However, the above two countries have dedicated more resources to countering Islamist-inspired terrorism than right-wing extremism (Budning, 2023).

Studies indicate that young individuals are more susceptible to radicalization than older demographics, primarily due to their lack of life experience and extensive engagement with social media platforms (Alava et al., 2017; Hassan et al., 2018; Miconi et al., 2023). Amidst our progressively social media-driven world, extremist organizations have demonstrated heightened proactivity in targeting young individuals through online platforms (Hutchinson, Amarasingam, Scrivens, & Ballsun-Stanton, 2021; Preston, 2023). As a result, youth radicalization through digital media has become an increasingly prominent issue in the West (Government of Australia, 2024).

While much attention has been devoted to exploring the dynamics and drivers of violent extremism from various perspectives, there remains an underexplored area in the literature regarding the voices and perspectives of young students (adolescents under 18) in the West, especially in Canada, on this pressing issue. Given that adolescents represent a crucial demographic vulnerable to radicalization and recruitment by extremist groups, as highlighted earlier, understanding their voices and experiences around this extremism is invaluable for informing effective prevention and intervention strategies. This empirical study seeks to address this urgent necessity by exploring Canadian secondary school^1^ students’ voices around violent extremism, aiming to understand their perceptions, concerns, and experiences regarding this critical issue. By doing so, this research seeks to uncover the underlying factors contributing to youth vulnerability to radicalization and violent ideologies. It also strives to understand the effectiveness of existing educational and community interventions. Ultimately, the study provides evidence-based recommendations to combat youth radicalization in Canada, fostering inclusivity, resilience, and proactive measures within schools and communities to address the root causes and prevent the spread of extremist ideologies.

By engaging directly with students in qualitative interviews, this study aims to answer these main research questions: 1. How do these young people conceptualize or understand violent extremism? 2. Are they curious about this issue, and if so, how do they seek information on it? 3. How do they perceive the role of education in preventing/countering violent extremism (P/CVE)? 4. To what extent do they feel their voices are heard in school regarding this critical topic and other relevant issues? Furthermore, this study pays attention to various influences, including social media, peer interactions, family dynamics, and educational environments, in shaping students’ understandings of and responses to violent extremism.

Overall, our data analysis yielded seven themes as follows:

• Most students have limited or misinformed views on violent extremism.
• The vast majority lack awareness of non-religious and far-right extremism.
• Discussions on violent extremism are notably absent in schools.
• Students are curious about the topic but often seek answers from social media.
• Muslim students face identity struggles due to misconceptions about extremism.
• Student reactions are mixed regarding the role of education in P/CVE efforts.
• While some feel heard, others share troubling experiences within schools.

## Theoretical Perspectives

### What is Extremism and Violent Extremism?

Desmond Tutu defines extremism as “when you do not allow for a different point of view; when you hold your own views as being quite exclusive; when you don’t allow for the possibility of difference” (quoted in Davies, 2009, p. 4). Then, violent extremism refers to the use or advocacy of violence to achieve political, ideological, or religious goals, often in defiance of democratic principles, pluralism, and human rights. The radicalization process that leads individuals or groups to violent extremism can be fueled by personal, social, or political grievances, with extremist ideologies providing a framework that justifies such violence (Horgan, 2008; Schmid, 2011).

Violent extremism can be religious, right-wing, or left-wing. Religious extremism is characterized by the use of violence in the name of religious beliefs. Groups like ISIS and Al Qaeda justify their actions through a misinterpretation of religious texts to legitimize acts of terror (Moghadam, 2009) or Christian nationalist groups such as Ku Klux Klan (KKK), Aryan Nations (AN), or many other religious groups who use their religious ideologies for the similar purposes (Schmid, 2011). Right-wing extremism, which can be religious or non-religious, is associated with xenophobia, racism, and nationalism. It also involves authoritarian, populist, and anti-democratic ideologies. These groups typically target ethnic minorities, immigrants, or political adversaries and are motivated by a perceived threat to group or national identity (Carter, 2018; McCauley & Moskalenko, 2008; Mudde, 2007). Islamophobia, which is anti-Muslim racism, can be seen as a by-product of right-wing political ideologies and right-wing extremism (Pickel & Öztürk, 2018). Right-wing extremism can also be connected to misogyny, homophobia, and transphobia, adding to the already complex nature of this phenomenon (Dier & Baldwin, 2022). Unfortunately, Canada has been a typical context for such manifestation of violent extremism. Some examples include the Montreal École Polytechnique massacre in 1989, which resulted in 14 deaths; the 2018 Toronto van attack (“Incel” inspired attack), which killed 11 people; the 2020 Toronto spa stabbings (another “Incel” inspired attack) that killed a young woman and seriously injured another; and the stabbings in gender-studies class at the University of Waterloo in 2023, seriously wounding three people.

We also witness violent ethnonationalism or separatist extremism. Followers of such extremism may seek political autonomy or independence based on ethnic identity through violence. This can include violent campaigns for secession, as seen in movements like the IRA (Irish Republican Army), ETA (Basque Homeland and Liberty), or the Khalistan movement (Kaldor, 2012). Less common globally, left-wing extremism, mostly non-violent compared to other forms of extremism, often arises from opposition to capitalism, globalization, imperialism, or state authority. Left-wing extremists want to replace current systems with communist or anarchist world orders (Jungkunz, 2019; Karmon, 2005). As an example, Antifa can be seen as a left-wing extremist movement rather than a formal group that is unlikely to engage in terrorist activities (Bray, 2017). However, certain left-wing groups, especially those driven by Communism or Marxism-Leninism, such as the Shining Path in Peru and the Naxalite-Maoist movement in India, have been labeled as terrorist groups as they used violence.

Closely relevant to extremism, radicalization refers to “diverse processes by which people come to adopt (extremist) beliefs that not only justify violence but compel it, and how they progress—or not—from thinking to action” (Borum, 2011, pp. 7–8). The causes behind such processes are discussed later in this section.

### The Potential Role of Education in P/CVE

Typically, efforts to counter violent extremism in North America have focused primarily on coercive and aggressive state measures characterized by the exertion of “hard power,” such as surveillance, policing, and military intervention. Meanwhile, the existing deradicalization programs do not focus on “the sources of radicalization but its symptoms” (Sklad & Park, 2017, p. 432). We acknowledge that integrating education into these strategies can offer a more holistic approach that addresses root causes, promotes critical thinking, fosters empathy, and builds resilience against extremist ideologies (Duckworth, 2024; Gereluk, 2023; Ghosh et al., 2016, 2017). By incorporating education initiatives alongside traditional enforcement methods, societies can work towards long-term solutions that tackle the underlying drivers of violent extremism and promote peaceful coexistence (Abu-Nimer, 2018; Ahmed & Shahzad, 2021; Danzell et al., 2018; Duckworth, 2024; Ghosh et al., 2017; Harris-Hogan et al., 2019).

Academic discussions on the role of school education in C/PVE have still been inadequate, given the complexity of the matter, which is intertwined with various other variables simultaneously (Duckworth, 2024; Korotayev et al., 2019). Yet, various extremist groups effectively leverage their own forms of education to disseminate and reinforce their radical ideologies within Western societies. Through multiple channels such as online forums, social media platforms, and clandestine gatherings, these groups indoctrinate young individuals by distorting historical narratives, promoting divisive rhetoric, and exploiting youth vulnerabilities (Hassan et al., 2018; Koehler, 2014). Their educational strategies often target disenfranchised communities, marginalized individuals, and youth who may be susceptible to manipulation. By providing a sense of identity, purpose, and belonging, extremist education can radicalize individuals and mobilize them toward violent action (Alava et al., 2017; Davies, 2016; Dilimulati et al., 2019). While education can counter violent extremism, we must also address its misuse by extremists, fostering critical thinking, resilience, and tolerance through counter-narratives and targeted initiatives.

### Factors Behind Radicalization

Borum (2011) warns against overemphasizing ideological factors, arguing that this approach can obscure the diverse array of other influences contributing to radicalization toward violent extremism. He suggests that involvement in violent extremism is a multifaceted process shaped by various factors that operate differently for individuals across different contexts and timeframes. To better analyze the reasons behind radicalization, a holistic focus on “the reciprocal interplay between… structural, social and individual factors” is needed (Sjøen, 2023, p. 236).

We align with these perspectives and acknowledge the existence of a wide range of push and pull factors that contribute to radicalization (Ghosh et al., 2016, 2017; Maraj-Guitard et al., 2021). By considering the complexity of this matter and the diverse influences at play, we can develop more nuanced approaches to P/CVE.

### A Key Factor—The Sense of Exclusion and Marginalization

Many scholars have unanimously highlighted the link between social exclusion or marginalization and radicalization (e.g., Bélanger et al., 2019; Ghosh et al., 2016, 2017; Hafez & Mullins, 2015; Jasko et al., 2017; Mucha, 2017; Sklad & Park, 2017). Some Canadian researchers have specifically examined radicalization within educational environments, suggesting that social isolation or marginalization may contribute to the radicalization of some young individuals (e.g., Dilimulati et al., 2019; Ghosh et al., 2023), yet there exist counterarguments. For instance, the Centre for the Prevention of Radicalization Leading to Violence (2016) finds that “the social positioning of individuals who undergo radicalization in Quebec is not, except in rare exceptions, that of individuals who are marginalized or who can objectively be said to be socially excluded” (34). We contend that such definitive conclusions should be approached with caution.

Thus, to address the possible sense of exclusion or marginalization experienced by students, we highlight the ethics of care framework developed by educationist Nel Nodding (1988, 1992), a moral theory emphasizing the importance of relationships, empathy, and compassion in ethical decision-making. Unlike traditional ethical theories, this perspective offers a relational and context-sensitive approach to ethics that prioritizes empathy, compassion, and responsiveness to the needs of others, especially the marginalized. It emphasizes the importance of ethical relationships, suggesting that fostering a culture of care in schools can be pivotal in addressing issues like radicalization by creating an environment where students feel valued and understood.

In conjunction with the ethical perspectives discussed above, we emphasize Albert Bandura’s moral disengagement theory (1999), which elucidates cognitive processes enabling individuals to justify harmful behavior while dissociating from moral agency, standards, and inhibitions. Bandura outlines mechanisms like moral justification, diffusion of responsibility, and dehumanization of victims that facilitate reinterpretation or justification of violent actions, enabling individuals to distance themselves from consequences. Bandura (1977) also underscores the role of observational learning in shaping behavior and attitudes, making it particularly relevant for understanding how exposure to social influences, such as peer groups and media, affects students’ perceptions of violence.

Adherence to care ethics principles may mitigate moral disengagement, as empathetic responses to ethical dilemmas prioritize compassion over self-interest. Individuals experiencing exclusion benefit from caring relationships, whereas their absence can exacerbate feelings of isolation, leading to moral distress and disengagement from ethical values, perpetuating a vicious cycle.

## Methods

The Research Ethics Board of McGill University approved this study. The ethical approval was renewed each year between 2017 and 2022. Our initial plan involved conducting a mixed-methods study comprising a large-scale survey, focus group discussions, and one-on-one interviews with secondary school students from Vancouver, Calgary, Toronto, and Montreal metropolitan areas. However, despite our earnest efforts to engage with various school boards to recruit research participants, we encountered significant challenges. Most school boards rejected our requests for access to their schools for data collection, citing a range of reasons. Some asserted that they did not perceive radicalization issues within their student body, despite our proposal stating that we would only be interested in knowing students’ knowledge or understanding of violent extremism. Others expressed concerns that our study could potentially harm their students, contradicting our intentions to provide support and prevention strategies. Shockingly, one English-language school board in Quebec suggested focusing on the French-language school system, reasoning that “most immigrants attend French schools.”^2^ This recommendation implies that immigrant students, particularly Muslims, are perceived as more susceptible to radicalization. While this response was deeply disappointing, we considered it a part of our research findings, suggesting that anti-immigrant and anti-Muslim biases related to violent extremism may be embedded within systemic frameworks in Canada, particularly in Quebec. For example, the province’s secularism law (Bill 21), which prohibits public sector employees from wearing religious symbols, has faced significant criticism for disproportionately affecting minority groups, especially Muslims. This policy, coupled with ongoing public debates regarding identity and secularism, may foster an environment where discriminatory attitudes toward Muslims are more likely to arise or be voiced. These contextual factors imply that the sentiment reflected in the school board’s response may not be an isolated incident but indicative of a broader trend.

Consequently, we resorted to using a snowball method to identify potential participants. The first participants were recruited through the secondary school teachers our team members knew. Then, we asked those participants to help us reach out to more people from their schools. Over the period from 2017 to 2022, we gathered data from 36 students through in-depth interviews while ensuring at least six students from each metropolitan area were included. Their ages ranged from 13 to 17. There were 32 individual and two group interviews (two participants per group). The former averaged 30–40 minutes, while the latter lasted about 50–60 minutes each. At the outset of our study in 2016–2017, our interview protocols were shaped by the scholarly and public discussions prevalent at the time, focusing primarily on students’ understanding of religious extremism. However, as the geopolitical landscape shifted, with the decline of ISIS and the rise of right-wing and other emerging forms of extremism, we adjusted our approach. Specifically, we replaced “religious extremism” with “violent extremism” to better reflect the evolving discourse. The research team sought feedback from experts in the field on the interview questions, allowing for an informed revision process.

Before COVID-19 lockdowns, interviews were conducted in-person (11 in 2017, three in 2018, and six in 2019), with the remainder held virtually (eight in 2021 and six in 2022). The complete set of interview questions can be found in Appendix 1. Since all participants were under 18, we obtained their assent forms and parental or guardian consent forms prior to the interviews. Due to the highly sensitive nature of our topic, we did not reveal participants’ personal information apart from their metropolitan areas, which are represented by using the first letter of each city. For example, we used “M” for Montreal, “T” for Toronto, “C” for Calgary, and “V” for Vancouver. Therefore, a secondary school student from Montreal would be coded as MS1 or MS2. A student from Toronto would be indicated as TS1 or TS2, etc. We chose not to explore disparities related to student gender, age, specific grade levels, socioeconomic, cultural, ethnic, and racial backgrounds, or dis/abilities for two key reasons: 1. Sample size constraints: Given the limited size of our sample, further differentiating these factors would not yield meaningful results or insights. 2. Privacy concerns: Due to the sensitive nature of our research topic, it was essential to prioritize the privacy and comfort of our participants. Introducing additional demographic categorizations could compromise their anonymity and deter open, candid responses. However, some participants voluntarily disclosed their Muslim background, providing valuable insights that were not part of the original research plan but enriched our findings.

The research team manually recorded and transcribed all interviews. Thematic analysis was employed to analyze the data, relying on an inductive approach (Grounded Theory) over deductive reasoning (Framework Analysis), as Maxwell (2013) outlined. Our analysis began with familiarization, immersing in the data through repeated reviews of transcripts and notes. Key statements were then systematically coded to capture patterns. The codes were then categorized into themes, reviewed, and refined for coherence and distinctiveness, ensuring they accurately reflected the data. Finally, the themes were integrated with existing literature to contextualize the findings within relevant and broader discussions.

## Findings

### Most Students Exhibit Superficial or Misinformed Viewpoints About Violent Extremism

We find that many students harbor a limited or misguided understanding of the concept of violent extremism. An illustrative example is a student who repeatedly uses the term “extreme” to characterize violent extremism yet struggles to provide deeper explanations. While recognizing the harmful and erroneous nature of such behavior, this student faces challenges articulating their understanding:

Interviewer: What is violent extremism, in your opinion?

TS3: Well, it's usually taking an issue or a problem of a group or a society and making it way too extreme and distracting or trying to find a solution to that extreme perception of that issue. It tends to be uncalled for; the actions that are extreme usually tend to be uncalled for; they tend to be born out of a misconception. They're often damaging, I guess. That’s pretty much all I have.

Echoing the above response, for MS9, “Violent extremism is like doing terrible acts like killing people for a cause that you take too far in a way that is too much, too exaggerated….” Surprisingly, a religious school student (TS4) (self-identified) had not heard of the terms “religious extremism” and “violent extremism” before participating in our study. For MS3, “it’s just basically hate crime attacks.” Similarly, in the eyes of TS5, it is all about “fear and hate.” MS12 thinks that “Damian Clairmont^3^ maybe had a certain illness or something.”

For MS6, violent extremism is “engraved in one’s personality...maybe from birth or even you develop it within one’s family or the way one grows up.” For MS9, “it is like drug addiction…” MS13 thinks the main reason behind Clairmont’s radicalization was his lack of knowledge of other religions except Islam, implying that Islam may have an intrinsic push factor towards radicalization. Participant CS7 defines it as “a strong dedication to a specific religious belief.” This interpretation implies that CS7 categorizes individuals deeply committed to their faith as extremists. Two other Calgary students (CS1 and CS2) have misguided knowledge about ISIS. “Is ISIS fighting against Islam?” asks CS1.

Meanwhile, we find that the majority of Calgary participants have not heard of the infamous case of Damian Clairmont, who went to Syria to join ISIS. For instance, CS6 says they “never heard about it (news about Clairmont) but heard about other countries like, I think, England.” Only CS8, CS9, and CS14 are aware of it, but they do not know much about it in detail. CS7 knows about the Montreal cases,^4^ though.

Only several students (five) demonstrate a somewhat reasonable understanding of the ideological underpinnings of violent extremism. For example, according to MS8, violent extremism “could be something that feels enticing or appealing but ultimately leads to behavior that contradicts ethical principles or societal norms.” In a similar vein, M11 articulates it as “people who have an idea they want to make it happen so badly that they use violence.” According to MS12, Violent extremism is “Violent actions that are motivated by a radical way of thinking.” MS2 also points out the “belief-driven” nature of violent extremism. MS7 shares some important triggers behind radicalization paths, such as personal tragedies and social exclusion.

### The Vast Majority are Ignorant of Non-religious and Right-wing Extremism

We find a general lack of awareness and knowledge among students of non-religious and right-wing extremist events/groups, revealing gaps in their understanding of the broader spectrum of violent extremism. One critical issue is that they tend to overemphasize Islamist extremism in response to the question of violent extremism. Meanwhile, many seem to be unaware of the existence of religious extremism in other religious groups such as Christian, Hindu, and Buddhist, and most are not aware of right-wing extremist groups and events.

For instance, the following student fixates on the Middle East while responding to our question on violent extremism.

Interviewer: What comes to your mind when you hear words like violent extremism?

TS2: What comes to my mind is war or some sort of like fighting, a war, and the stuff that's going on in the Middle East right now (June 2022).

Interviewer: Middle East, so no other places other than the Middle East?

TS2: Well, that's where most of the stuff that's happening right now is. There is a lot of war and violence going on in the Middle East right now.

MS6 cannot give examples of violent extremist events apart from “the Twin Tower incident,” meaning the 9/11 terrorist attacks. MS9, too, primarily highlights Islamist extremism.

MS9: When I hear violent extremism, firstly, [I think of] the Islamic individuals who take actions in the name of Islam. Secondly, [I think of] communism in certain countries. These are the two main associations I make with the concept of violent extremism.

While seeing communism as a form of violent extremist ideology can be reasonable, this is not an established perspective within academia. For MS5, without a religious context, it is difficult to understand violent extremism. “It is kind of inevitable,” declares MS5. TS2, TS7, and VS3 have not even heard of right-wing extremism. Only a few students (five) can identify specific examples of non-religious, right-wing or left-wing extremism. For instance, TS3 mentions hearing about groups like the Proud Boys and the KKK. MS1 points out Antifa and La Meute in Quebec. MS4 shares that ISIS, along with Atomwaffen Division, Northwest Front, and the Base, was introduced by one of their teachers. VS1 says they learned about the KKK through YouTube videos and found it intriguing how some individuals could be “so closed off to differing perspectives.”

Delving deeper into the topic, MS5 provides additional insights:

Interviewer: Do you think non-religious extremist groups exist?

MS5: Yes. The thing is, a lot of groups that say they are not religious sometimes have religious beliefs in them. For example, Proud Boys have pretty misogynistic and sexist views. They would say women should act like this because of the idea that the Bible says [so]. But some extremist ideologies like racism have less to do with religion; it has more to do with power.

While the above student rightly identified the misogynistic and sexist aspect of Proud Boys, no single student could point out individual attacks by Incels or incidents triggered by misogyny, homophobia, or transphobia that happened on Canadian soil, which we highlighted earlier.

### Students Note a Distinct Lack of Discussions on Violent Extremism in Their Classrooms

Most students stress the lack of the opportunity to discuss topics around violent extremism in class. For example, MS2 shares that they “have talked about a lot of topics this year. Some topics are new, like genetic modification … but [they] don’t think [they] have covered specifically extremism.” CS11 and TS2 also depict the same reality, exposing that even some extremist events recently happened in Canada were omitted by their teachers. “Many schools don’t really talk about this topic, or they refuse to even mention it, and if you do, I guess you get into trouble,” expresses TS9. Similarly, CS5 says: “we definitely do not talk about that in class; I don’t think anyone has the comfort to do so.” MS6 confirms that only one of their teachers talked about the 9/11 terrorist attacks briefly in one class during the 20th anniversary of the event. CS8 even declares that “this is the only conversation (meaning our interview) [during which they] have talked about radicalization.”

A few others (seven) acknowledge discussing issues related to violent extremism, but these conversations often remain superficial, akin to news briefs. For instance, CS8 reflects on social studies classes, noting discussions limited to surface-level news items about violent events without delving into the psychology or root causes of radicalization. Similarly, CS14 shares an experience where the Paris attack was mentioned in class but “not really discussed.”

According to VS2, some teachers tend to address historical extremist events rather than recent occurrences, such as “talks on past ideologies like fascism and communism.” MS8 also confirms a focus on historical figures like Adolf Hitler. MS12 observes that conversations about extremism typically arose in their school only after relevant events occurred, like the London truck attack in 2021. MS4, too, points out limited discussions on extremism, primarily initiated by ethics or history teachers and often prompted by specific events rather than as part of a comprehensive curriculum. CS6 notes the sole emphasis by teachers on condemning such issues rather than providing “any explanation or how to deal with them.”

Only three students report gaining meaningful knowledge of violent extremism from their teachers. For instance, TS9 recalls discussing various forms of violence in their school, including violent extremism and racism, especially following notable incidents like the London truck attack in June 2021. Two other students express that their teachers who cover ethics, history, or social studies frequently addressed topics related to violent extremism. For example, MS5 recalls discussions on ISIS and Al Qaeda and current events such as the Quebec Mosque shooting. CS7 shares the same reality and a desire for more emphasis on preventive measures rather than explaining what happened and why.

That said, CS14 raises concerns about students being left to seek information independently. “It feels like we’re supposed to know these things on our own and learn about them in our own time,” the student states. MS7 also points out that “young people are influenced by the Internet and social media regarding extremism while there is not much guidance from their schools.”

### Students are Inquisitive About This Topic but Often Turn to Alternative Sources, Especially Social Media, for Help

Despite a significant lack of classroom discussions on violent extremism, students consistently exhibit a strong curiosity about the topic. Their inquiries span a range of issues, including the root causes and motivations behind extremist ideologies, the tactics employed by extremist groups, and the broader social impact. These questions demonstrate a genuine desire to explore and better understand this complex phenomenon.

For example, TS1 questions why individuals join extremist groups despite being aware of “their unethical and inhumane actions.” This perspective reveals a misconception—that violent extremists are not morally disengaged. Similarly, MS13 wonders how extremists manipulate others into supporting their cause, while TS6 wants to explore the psychological factors that drive individuals to view violence as the only path to achieving their goals, whether religious or otherwise. MS4 seeks to uncover the hidden motives of extremist group leaders, suggesting “a search for power” as a likely reason. MS12 feels confused about the radicalization process, particularly in religious contexts, where ideology is typically associated with goodness. “But in religion, everything that is said is supposed to be good,” MS12 notes. VS6 echoes this sentiment, grappling with the contradiction of religious belief and violence committed in its name. MS5 expresses bafflement about how individuals can overlook atrocities caused by extremist organizations like “ISIS, Al Qaeda, and KKK” and still perceive inherent good within them. CS3 brings attention to social media’s role in perpetuating biases against Muslims and questions how to address this issue effectively.

While a few students, such as TS10 and MS11, express confidence in their understanding of violent extremism, the majority reveal knowledge gaps and uncertainties. Due to the lack of classroom discussions, many students seek information elsewhere. CS4, for example, mentions discussing these issues informally with friends: “Not in class, but with friends I do; we talk about it a lot; we talk about it all the time. I am really interested in such issues.” Others, like CS14, rely more on the Internet and social media: “We don’t discuss it at home or school, but I can learn about it through social media—Facebook, online news, and so on….”

These narratives underscore the urgent need for schools to provide students with accurate and reliable information on violent extremism. Without adequate guidance from educators, students turn to informal sources or online platforms, where misinformation is rampant, and extremist groups actively target vulnerable youth for recruitment. To address this issue, schools must proactively equip students with the skills to navigate online space critically and responsibly.

### Muslim Students Appear to Experience Significant Identity Crises

We did not require participants to disclose their religious identities or affiliations during interviews. Instead, insights into their religious backgrounds emerged organically through their narratives. Notably, six students voluntarily shared their Muslim identity while discussing the topic, and their reflections highlight a critical theme that deserves attention. We find that these students experience confusion and a sense of misrecognition regarding their religious identity in relation to violent extremism. They face challenges navigating their Muslim identity amidst prevailing stigma and misconceptions surrounding extremism and Islam. This underscores the need for greater recognition and cultural sensitivity in educational settings and the broader society.

For instance, TS8 reflects on the association between violent extremism and Islam, which they find deeply puzzling given their peaceful interpretation of the religion.

Yes, I have puzzles, for me, I am Muslim, and then one day is like violent extremism is related to Islam, for example, the Taliban and ISIS and all that. Right? They also can claim to be Muslim, and they are also Sunni Muslims like I am… But the action they do and the way they view Islam is very different from how I do. So, that really puzzles me, how they extract meanings from a peaceful religion.

Similarly, TS5 raises concerns about the misrepresentation of Islam in the media, where violent acts committed by individuals claiming to be Muslims contribute to the misconception that Islam is inherently violent. CS3 also highlights the widespread anti-Muslim biases on social media and their negative impact on perceptions of violent extremism, emphasizing the difficulty of countering these narratives. CS4 recounts a distressing personal experience of discrimination. “I went to McDonald’s, and a girl came up and started saying, ‘Oh, you guys are from ISS bla bla bla for no reason….’” CS4 also shares incidents where their siblings faced bullying and discrimination at school due to being visibly Muslim and having sensitive Arabic names, such as “Osama,” which lead to his withdrawal from the institution. “Even my sisters, you know, don't wear their hijabs because they are scared,” adds CS4.

At the same time, some Muslim students (three) assert their agency in challenging these misconceptions. For instance, MS13 stresses the urgent necessity to dissociate Muslims from radicalism and advocates for rejecting harmful stereotypes. While echoing the same, CS14 strongly denounces that groups like ISIS represent the broader Muslim community. CS5 passionately expresses:

I talk to my parents about this type of extremism; I am from Syria. I have been here for five years now. We moved because of the war, and my parents have been discussing that topic all the time because you are part of it. If you are part of Islam, you need to defend yourself when people judge you as a minority. I will have to stand up by myself and tell them why I am not part of ISIS, who I am as a Muslim, what my values mean as opposed to theirs (ISIS’s), and so that’s definitely something [I have to do] as part of my family; we talk about it all the time.

### Students Hold Diverse Opinions on the Role of Education in P/CVE

“The less educated someone is, the more they are likely to join these extremist groups,” asserts VS6. VS1 offers similar points:

100 percent! I think education is such a huge part of someone’s development in becoming a good person. I spend like 30 hours a week at school. And yet, the experiences I have in school will translate into what I do outside of school. So, I think if you have a really positive school education experience, your life is probably going to be better.

TS4 also notes that the time students spend at school is a crucial factor, while VS4 credits their school for teaching values like acceptance and empathy. MS9 and VS5 stress that education can address broader societal issues, including violent extremism, and MS11 values informal learning alongside classroom instruction.

However, some students point to the need for more targeted education on violent extremism. For example, “Yes, if people (teachers) *know* [emphasis] what they are talking about and they have invested in their work,” says MS13. In a similar vein, MS4 highlights the potential of such education, saying, “because the students might not be aware of the tactics extremist groups use to pull people into their organizations.” Two other students echo these viewpoints by noting that “it should be taught properly” (TS9) and “more directly” (CS10). TS1 laments the absence of such education in schools. TS8 suggests incorporating such discussions into school assemblies, and TS6 believes such conversations should be student-driven rather than mandated by schools.

Interestingly, VS3 stresses the importance of monitoring extremist circles, particularly “in communities with fundamentalist Muslim influences.” While the student’s perspective on monitoring is understandable, we sense an overemphasis on Islam, as discussed earlier.

However, CS6 feels schools fall short in this regard. Although TS1 acknowledges the potential for education to prevent extremism, the student also views that “if someone really wants to do something...I don’t think they’re gonna listen to their school.” “I don’t think reading from a textbook can do any good,” says CS12. MS3 also expresses uncertainty, stating:

I have no clue about it. No idea … I think sometimes things could change like that, but after completing education, sometimes the person takes the wrong path. That's all I know.

TS2 has the same skepticism and underscores the autonomy of individuals to form their own opinions and paths, suggesting that school teachings may not always influence student behavior.

We also sought to gauge how students perceive their own schools in fostering resilience against violent extremist ideologies. Again, our findings suggest varying levels of optimism. For example, MS11 expresses confidence in their school’s ability, citing its comprehensive approach to education. TS9 appreciates their school’s efforts in organizing seminars and lectures to educate students about “a lot of issues associated with extremism.” TS11 sees potential in their psychology classes to help students become resilient against radicalization. MS9 highly regards the impact of history and citizenship classes, while MS10 shares a project they did in religious culture class that helped them understand radical groups better. MS10 reflects:

I wouldn’t know about other schools, but at my school we were lucky because in the course religious culture, I made a project about radical groups. The teachers put us in pairs and each team had to research about a radical group around the world. I believe that it allows us to better understand radical groups and where they come from and not just say that radical groups are bad.

However, some students (seven) desire their schools to do more. For instance, MS4 acknowledges his school’s multicultural environment as a positive factor but feels the school fails to address extreme ideologies. MS1 argues that their teachers “have to understand extremist propaganda and its psychological impacts.” MS13 stresses the importance of inviting experts on terrorism to inform students regularly, as their teachers lack expertise in the field. For CS2, the short answer is “maybe it (their school) is helpful.” According to MS5, their school “isn’t open to extremist views (meaning a lack of discussions of such views), but it isn’t very preventive either.”

CS4 finds it challenging to talk about violent extremism with their teachers while recounting an incident in a social studies class where the student raised this topic. This led the school principal to contact the student’s mother, expressing concern that CS4 might be at risk of radicalization. Despite this, CS4 remains committed to engaging in conversation on the subject, stating, “That’s why I wanted to come to talk to you guys (the research team).” Similarly, TS2 shares a troubling observation, noting, “There are indeed a few teachers who do not demonstrate respect towards all students. There have been incidents like teachers being involved in racism and other stuff.” Such patterns demonstrate significant challenges that could undermine P/CVE efforts.

### Although Students Generally Feel Heard, Some Report Troubling Experiences

Exploring if the students feel their voices are heard in school regarding various controversial matters reveals that the majority express a sense of care and attention from teachers. MS12 highlights the environment where teachers actively seek students’ opinions, fostering an inclusive culture. “The teachers are always kind of asking about the opinions of the students,” expresses MS12. Similarly, CS2 praises the open atmosphere at their school, where diverse perspectives are respected. “I feel no one is afraid to speak about their opinion because here teachers are really understanding,” notes CS2. In the same vein, CS1 has confidence in seeking support from teachers and counselors when facing problems. VS4 shares a positive encounter discussing alternative viewpoints on school shootings in a social studies class, appreciating the nuanced discussions led by their teacher. VS5 finds increased freedom in their secondary school to express diverse opinions compared to their elementary school.

Despite these positive experiences, there are instances where students feel unheard, especially on sensitive topics like violent extremism. As an example, TS9 reflects on varying levels of openness across schools regarding the topic. TS5 expresses frustration at the lack of platforms for discussing “personal opinions on such global issues in class.” Meanwhile, MS7 and TS2 show their hesitancy in raising such issues in class due to their teachers’ biases or one-sided perspectives. MS5 also admits never approaching teachers, indicating a lack of confidence. MS3 highlights the perceived disinterest of teachers, while TS6 self-censors “to avoid causing discomfort to others.”

CS3 points out how a teacher prioritizes avoiding conflict over engaging in a genuine conversation with students. “If she (the teacher) does not agree with one person on violent extremism, she just tries to go with the flow like yeah, ‘you are right, yeah exactly’,” notes CS3. TS4 suggests that ensuring every student feels heard is challenging for their teachers, “particularly in large class settings where individual engagement may be limited.”

Here, we also emphasize MS13’s very negative experience as it may reveal some systemic issues affecting some students with minority backgrounds, especially Muslims. MS13 narrates:

I think that my teacher is not my friend; I cannot talk to [them] about topics like that. I personally think that they just want to dictate the course and go back home. It is like that. Most teachers tell us: “if you have a problem, come and talk to me,” but as you keep growing up, you start to see that teachers just want to dictate their course, get paid, and leave, and that is it. Even if you have a problem, you are not inclined to get help from teachers... Something that discourages youth is when there's a conflict between a student and a teacher. For example, this year, my friend and I were insulted, etc. by the English teacher, but we really had not done anything. He targeted us due to racism; he targeted us all the school year because we are Muslim. My friend and I went to the principal’s office to talk to him about it, and he told us: “Yeah, but he is a teacher...” Ok, he is a teacher, so he can keep doing it?

It is hugely disappointing to listen to the above responses by MS13. The perceived lack of rapport between MS13 and their teachers and school principal, coupled with feelings of being unheard or discriminated against based on their racial and religious identity, may underscore systemic challenges faced by students from minority backgrounds, particularly those of Muslim descent. Highlighting the above anecdote and its implications is particularly important and relevant because feeling marginalized or excluded can become a push factor leading to youth radicalization, as discussed earlier.

## Discussion

There exist some empirical studies on teachers’ experiences in engaging in topics related to violent extremism in Finland (Vallinkoski & Benjamin, 2023; Vallinkoski et al., 2022), Canada (Dilimulati et al., 2019, 2024), Estonia (Maiberg & Kilp, 2022), Norway (Sjøen, 2024; Sjøen & Mattsson, 2019), and Kenya (Breidlid, 2021). However, limited studies focus directly on young students’ voices around violent extremism, probably due to their young age and the sensitive nature of the subject. Among the few studies, Benjamin et al. (2021) conducted a large-scale survey (both quantitative and qualitative) involving 3617 students from Finnish upper-secondary schools and vocational institutions (ages 16–20). One key aspect of the survey asked about students’ evaluations of the knowledge they received in school on topics such as “religions and worldviews,” “extremism and terrorism,” and “peace promotion and conflict resolution.” Interestingly, 40% of the students considered the knowledge provided about extremism and terrorism to be inadequate. This contrasts with findings from our study, where most participants expressed similar concerns.

Eldor et al. (2022) explored student resilience to extremism in Norway by analyzing survey data from 233 upper-secondary students, primarily from urban areas. Their study aimed not to measure students’ understanding of violent extremism but instead focused on factors that foster social cohesion. They used multiple items under three main themes: (1) the perception that the school treats pupils equally no matter their social backgrounds, (2) the perception of the school and its employees as attentive and proactive in meeting pupils’ anger resulting from social and political issues, and (3) the presence of mutual respect. An item reads, “I can share my opinions with school staff even if they disagree with me.” The study finds that resilience against extremism can be nurtured by fostering an egalitarian school environment (p. 8).

The above two studies demonstrate the importance of an inclusive and supportive school environment in addressing extremism, with which our study totally resonates. Our findings also suggest an urgent need for a formal and comprehensive education on violent extremism. More specifically, we call for evidence-based approaches that foster content knowledge, critical thinking, and resilience against violent extremism. The emphasis should be on meaningful discussions of this topic, rather than expecting teachers “to deploy vigilant surveillance against students considered to be at risk of becoming a terrorist” as rightly pointed out by Sjøen (2021, p. 309). The youth’s overreliance on online platforms for information on violent extremism, as revealed in our study, further underscores the need for such initiatives. Meanwhile, research indicates that solely being overexposed to an online environment (for seeking any content) could make young people more vulnerable to radicalization. For example, Miconi and colleagues’ (2023) recent study reveals a positive association between increased online social interactions and the heightened endorsement of violent radicalization.

In one of our earlier studies related to this research, which was based on the data collected from 2017 to 2019, we found that many students were quite familiar with the notion of religious extremism (Dilimulati et al., 2019). Our current research, further enriched by the interviews conducted in 2021 and 2022, reveals that the subsequent student cohorts may have developed a more comprehensive and nuanced understanding of violent extremism following the rise of right-wing and some new forms of violent extremism. However, we still consider this progress to be limited as our participants are almost unanimously ignorant of the violent extremism triggered by misogyny, Incel ideology, homophobia, transphobia, etc.

Our findings also point to the importance of promoting emotional well-being among students. This resonates with Miconi and colleagues (2022) study that investigates the associations between a positive future orientation, the presence of and search for meaning in life (which is directly connected to a sense of belonging and inclusion), and support for VR (violent radicalization) in a diverse sample of a total of 3100 college students in Quebec. Their findings revealed that a positive future orientation and a higher presence of meaning in life were negatively associated with support for VR. Furthermore, the negative correlation between the sense of purpose in life and support for VR was more pronounced among students with a more optimistic outlook for the future.

Thus, educational institutions should provide more initiatives that foster positive and ethical personal characteristics among students and create an environment conducive to promoting students’ sense of belonging and emotional well-being. Our findings highlight the centrality of relational care in addressing violent extremism, aligning with Noddings’ (1988) ethic of care, and Bandura’s perspectives on morality (1999) and social learning (1977). As our participants suggest, trust and inclusive school environments foster protective relationships against feelings of alienation. Observational learning can expose students to narratives enabling moral disengagement (Bandura, 1999), while promoting critical reflection and ethical awareness in schools disrupts this process. Therefore, by addressing the ethical and cognitive dimensions, educators can empower students to resist harmful ideologies and develop resilience against extremism. This, in turn, will help young people strengthen their moral agency, empowering them to dismantle extremist ideologies and resist their influence.

There exist multiple limitations to this study. First, the scope of our findings is limited to the four metropolitan areas in Canada, which may affect their broader generalizability. Second, with a sample size of only 36 participants, the range of viewpoints captured might not fully represent the wider student population. Third, between 2017 and 2022, significant shifts occurred in North America, which must have affected participant responses. While acknowledging this possibility, we could not analyze the effects of various events and turning points in detail. Future studies could adopt mixed methods to overcome these significant limitations, combining large-scale surveys with more interviews and focus groups. Expanding the research to cover more diverse geographic regions across Canada will also improve the depth and generalizability of the results. Meanwhile, longitudinal data collection may not be very suitable for such topics, which are fast evolving.

### Recommendations

(1) Schools should incorporate education on violent extremism into their curricula. Evidence-based resources, such as those from the Institute for Strategic Dialogue, can help tailor materials to different age groups. Studies like Horgan and Braddock (2010) suggest that factual knowledge about extremist ideologies enhances students’ understanding. Meanwhile, given students’ tendency to seek information online, which is fraught with disinformation, it is crucial to guide them through this complex issue through formal education. This can be achieved through diverse resources, such as expert guest lectures, documentaries, and podcasts promoting interactive learning.
(2) Educators need the appropriate knowledge and tools to engage students in open discussions to challenge extremist narratives. To ensure this, government agencies and local educational institutions should actively invite subject-matter experts to train teachers on effectively addressing violent extremism in the classroom.
(3) Schools should enforce policies promoting diversity and respect, specifically addressing issues related to religious and cultural differences. Cultural competency training and anti-discrimination measures for educators can create a more inclusive learning environment. Initiatives like multicultural clubs and interfaith dialogues can improve social cohesion and reduce the risk of youth radicalization. This is akin to what Dianne Gereluk (2023) suggests: a whole-school approach that fosters belonging, inclusivity, and safe environments across the school community.
(4) Schools must implement targeted support, such as counseling programs or information sessions hosted by experts, for Muslim students facing discrimination or identity challenges due to misconceptions about Islam. Raising awareness within the school community about the unique challenges faced by Muslim individuals is equally important.
(5) Schools should prioritize fostering emotional well-being and resilience among students by promoting values such as empathy, ethics, and belonging. As Noddings (1988) suggests, this approach can help students feel valued, improve their emotional health, and strengthen their resilience against extremist ideologies. By empowering students with moral agency, schools can play a pivotal role in building a more peaceful and inclusive society (Bandura, 1999).

## Conclusion

In our research, we have explored the voices of Canadian secondary school students regarding the sensitive issue of violent extremism. Through our analysis, several key themes have emerged, shedding light on the complex nature of this issue and its ramifications for various stakeholders. We find that many students hold limited and misinformed views on violent extremism, indicating a significant gap in relevant discussions within Canadian secondary schools. A notable concern arises from the tendency to directly associate Islam with violent extremism while exhibiting a severe lack of knowledge of non-religious or right-wing extremism. Despite their curiosity, schools often fail to provide students with adequate and structured knowledge, leaving them to turn to informal sources, especially social media, for information, which may be unreliable and misleading. Given extremist groups’ pervasive use of the Internet and social media to facilitate their missions (Gaudette et al., 2022), it is imperative to provide students with better guidance in navigating online content to prevent misinformation and its harmful influences.

While student opinions on the role of education in P/CVE efforts vary, some express skepticism around the effectiveness of education in this matter, and some share troubling experiences of feeling unheard or marginalized within the school. Our research also reveals distinct identity crises and stigma faced by Muslim students, some reporting instances of marginalization and discrimination stemming from prevailing discourses associating Islam with violence. Alarmingly, some of these encounters involve educators, underscoring the urgent need to foster inclusivity and cultural sensitivity within educational environments.

Based on these findings, this research recommends incorporating comprehensive education on violent extremism into school curricula to develop students’ critical thinking and resilience. Given student overreliance on online platforms, schools must guide students toward credible sources to prevent exposure to disinformation. Teacher training should also be prioritized, ensuring educators are fully equipped to engage in open discussions and challenge extremist narratives. Meanwhile, schools must implement inclusive policies addressing religious and cultural diversity through cultural competency training and anti-discrimination measures. Targeted support for Muslim students, alongside open dialogue on religious diversity, is essential to foster understanding. Finally, promoting emotional well-being through an ethics of care framework will help students feel valued and build resilience against extremist ideologies, ultimately fostering a more inclusive society.

## Notes

1 In most Canadian provinces and territories, secondary school, also known as high school, covers grades 8, 9, or 10 through grade 12. However, in Quebec, secondary school starts at grade 7 and ends at grade 11.

2. Under Quebec’s Charter of the French Language (Bill 101), immigrant children are generally required to attend French-language public schools, and they are not automatically eligible to enroll in English-language public schools. This policy is designed to promote and preserve the French language and culture in Quebec.

3. Before starting interviews, we asked participants to watch a YouTube video about Damian Clairmont, a radicalized young man from Calgary, Canada, who went to join ISIS in 2012. He was killed in 2024 in Syria. For more information about the man, see https://calgaryherald.com/news/local-news/more-needs-to-be-done-to-prevent-homegrown-extremism-anti-radicalization-experts; the link to the video: https://www.youtube.com/watch?v=dFAKam_olzw.

4. In early 2015, at least six young Quebecers from Montreal left the country to join militants in Syria. For more information, see https://www.cbc.ca/news/canada/montreal/syrian-jihadists-believed-to-have-recruited-6-young-quebecers-1.2973089.

## Appendix 1

### (Key Interview Questions)

(1) Christianne Boudreau is Damian Clairmont’s mom. She describes his childhood and conversion in this video: https://www.youtube.com/watch?v=QBZSHAXhUyI
  (a) What are your thoughts?
  (b) What do you think was going on through his mind when he went to Syria?
  (c) What are some of the reasons that would lead him to become radicalized and closed to other ideas?
  (d) What do radical actions lead to? Do these actions have a goal in mind?
  (e) What kind of role does religion play in a discussion on radicalism?
(2) Discuss their understanding of violent extremism
  (a) When you hear the words violent extremism, what do you think?
  (b) How would you define the term “violent extremism”?
  (c) Is there something about violent extremism that puzzles you? If so, what is it?

The behaviors listed below have been identified as early signs of radicalization by the French government. What do you think about this list? The following behaviors can be early signs of radicalization. If several are observed, the family and immediate circle should be alerted.

• Sudden break with the family and long-standing friendships.
• Sudden drop-out of school and conflicts with the school.
• Change in behavior relating to food, clothing, language, and finances.
• Changes in attitudes and behavior towards others: antisocial comments, rejection of authority, refusal to interact socially, signs of withdrawal, and isolation.
• Regular viewing of Internet sites and participation in social media networks that condone radical or extremist views.
• Reference to apocalyptic and conspiracy theories.

Source: https://www.stop-djihadisme.gouv.fr/

(3) Education
  (a) Do you think education you receive at school can do anything about violent extremism?
  (b) Fauzia Duale Virtue is part of the Somali community in Toronto. She struggled with her multiple identities as a refugee and now works with Somali youth to help them navigate their way through similar identity issues. Watch: https://www.youtube.com/watch?v=7HzBqLUqlR4.
    (i) In the first part of this video, Fauzia talks about giving students opportunities to voice their opinions and ideas about violent extremism. Do you think your voice is heard in school? Why or why not?
    (ii) In the second part of the video, Fauzia talks about humanizing people from different backgrounds. Do you think the lessons you are taught in class or the relationships you have with teachers and counselors at your school can teach you how to humanize, or respect, different people in the way Fauzia discussed?
  (c) Do you think school and education in general can make a difference in how people understand violent extremism?

## References

[bibr1-00220574251318312] Abu-Nimer M. (2018). Alternative approaches to transforming violent extremism: The case of Islamic peace and interreligious peacebuilding. Berghof Foundation. Available at: https://cabar.asia/wp-content/uploads/sites/8/2019/01/dialogue13_Abu-Nimer_lead.pdf

[bibr2-00220574251318312] Ahmed Z. S. Shahzad R. (2021). The role of peace education in countering violent extremism in Pakistan: An assessment of non-governmental efforts. Conflict, Security and Development, 21(3), 199–222. 10.1080/14678802.2021.1943150

[bibr3-00220574251318312] Alava S. Frau-Meigs D. Hassan G. (2017). Youth and violent extremism on social media: Mapping the research. UNESCO. Available at: https://unesdoc.unesco.org/ark:/48223/pf0000260382

[bibr4-00220574251318312] Bandura A. (1977). Social learning theory. Prentice Hall.

[bibr5-00220574251318312] Bandura A. (1999). Moral disengagement in the perpetration of inhumanities. Personality and Social Psychology Review, 3(3), 193–209. 10.1207/s15327957pspr0303_3 15661671

[bibr6-00220574251318312] Bélanger J. J. Moyano M. Muhammad H. Richardson L. Lafrenière M. A. K. McCaffery P. Framand K. Nociti N. (2019). Radicalization leading to violence: A test of the 3N model. Frontiers in Psychiatry, 10(42), 42. 10.3389/fpsyt.2019.00042 30853917 PMC6396731

[bibr7-00220574251318312] Benjamin S. Salonen V. Gearon L. Koirikivi P. Kuusisto A. (2021). Safe space, dangerous territory: Young people’s views on preventing radicalization through education—perspectives for pre-service teacher education. Education Sciences, 11(5), 205–218. 10.3390/educsci11050205

[bibr8-00220574251318312] Bjorgo T. Jacob A. R. (2019) Extreme-Right Violence and Terrorism: Patterns, and Responses (8). ICCT Policy Brief. https://goto.now/w43iJ

[bibr9-00220574251318312] Borum R. (2011). Radicalization into violent extremism I: A review of social science theories. Journal of Strategic Security, 4(4), 7–36. 10.5038/1944-0472.4.4.1

[bibr10-00220574251318312] Bray M. (2017). Antifa: The anti-fascist handbook. Melville House.

[bibr11-00220574251318312] Breidlid T. (2021). Preventing violent extremism in Kenyan schools: Talking about terrorism. Journal for Deradicalization, 27, 71–107. https://hdl.handle.net/11250/2996879

[bibr12-00220574251318312] Budning K. (2023). The parallel threat: Political framing and right-wing extremism in Canada and the United States. TSAS Research Brief. Available at: https://goto.now/a66jd

[bibr13-00220574251318312] Carter E. (2018). Right-wing extremism/radicalism: Reconstructing the concept. Journal of Political Ideologies, 23(2), 157–182. 10.1080/13569317.2018.1451227

[bibr14-00220574251318312] CBC . (2022). White supremacy is a 'harsh reality' in Canada, says public safety minister. CBC. Available at: https://goto.now/ANmj1

[bibr15-00220574251318312] Centre for the Prevention of Radicalization Leading to Violence . (2016). Analytical report– radicalization leading to violence in Quebec schools: Issues and perspectives. Centre for the Prevention of Radicalization Leading to Violence. Available at: https://info-radical.org/wp-content/uploads/2016/08/rapport-cprlv.pdf

[bibr16-00220574251318312] Chen S. (2024). Far-right political extremism and the radicalisation of the anti-vaccine movement in Canada. In Lewis M. Govender E. Holland K. (Eds.), Communicating COVID-19 (pp. 303–323). Palgrave Macmillan. Available at: https://goto.now/ojtnT

[bibr17-00220574251318312] Danzell O. E. Yeh Y. Y. Pfannenstiel M. (2018). Does education mitigate terrorism? Examining the effects of educated youth cohorts on domestic terror in Africa. Terrorism and Political Violence, 32(8), 1731–1752. 10.1080/09546553.2018.1506336

[bibr18-00220574251318312] Davey J. Hart H. Guerin C. (2020). An online environmental scan of right-wing extremism in Canada. In ISD global. Available at: https://goto.now/7DVYP

[bibr19-00220574251318312] Davies L. , (2009). Educating against extremism: Towards a critical politicisation of young people. International Review of Education, 55(2-3), 183–203. 10.1007/s11159-008-9126-8

[bibr20-00220574251318312] Davies L. (2016). Security, extremism and education: Safeguarding or surveillance? British Journal of Educational Studies, 64(1), 1–19. 10.1080/00071005.2015.1107022

[bibr21-00220574251318312] Dier A. Baldwin G. (2022). Masculinities and violent extremism. International Peace Institute and UN Security Council Counter-Terrorism Committee Executive Directorate. Available at: https://unoct-communities-test.rhone.un-icc.cloud/system/files/2022-12/masculinities-and-ve-web.pdf

[bibr22-00220574251318312] Dilimulati D. M. Dhali H. H. Ghosh R. (2024). Talking about violent extremism: Experiences of Canadian secondary school teachers in four metropolitan areas. Journal for Deradicalization, 2(40), 72–113. https://journals.sfu.ca/jd/index.php/jd/article/view/955

[bibr23-00220574251318312] Dilimulati M. Dhali H. H. Ghosh R. (2019). The radicalization of youth in the West – how can Canadian teachers effectively approach this issue in their classrooms? In Jule A. (Ed.), The compassionate educator: Understanding social issues in Canadian schools (pp. 229–250). Canadian Scholars Press.

[bibr24-00220574251318312] Duckworth C. L. (2024). The role of schools in preventing violent extremism: From policing to pedagogy. Journal of Peace Education, 21(3), 382–401. 10.1080/17400201.2024.2314307

[bibr25-00220574251318312] Eldor D. S. Lindholm K. Chavez M. H. Vassanyi S. Badiane M. O. I. Yaldizli K. Frøysa P. Haugestad C. A. P. Kunst J. R. (2022). Resilience against radicalization and extremism in schools: Development of a psychometric scale. Frontiers in Psychology, 13(1), 980180. 10.3389/fpsyg.2022.980180 36438395 PMC9685524

[bibr26-00220574251318312] Gaudette T. Scrivens R. Venkatesh V. (2022). The role of the internet in facilitating violent extremism: Insights from former right-wing extremists. Terrorism and Political Violence, 34(7), 1339–1356. 10.1080/09546553.2020.1784147

[bibr27-00220574251318312] Gereluk D. (2023). A whole-school approach to address youth radicalization. Educational Theory, 73(3), 434–451. 10.1111/edth.12581

[bibr28-00220574251318312] Ghosh R. Chan W. A. Manuel A. Dilimulati M. (2017). Can education counter violent religious extremism? Canadian Foreign Policy Journal, 23(2), 117–133. 10.1080/11926422.2016.1165713

[bibr29-00220574251318312] Ghosh R. Manuel A. Chan W. Y. A. Dilimulati M. Babaei M. (2016). Education and security: A global literature report on countering violent religious extremism (CVE). Tony Blair Institute for Global Change. [Online].

[bibr30-00220574251318312] Ghosh R. Tiflati H. Dhali H. Dilimulati M. Chan W. Y. A. (2023). Social-ecological portrait of two women from Montreal who joined ISIS. Contemporary Voices: St Andrews Journal of International Relations, 5(1), 1–30. 10.15664/jtr.1626

[bibr31-00220574251318312] Government of Australia . (2024). Five eyes insights: Young people and violent extremism: A call for collective action. Government of Australia. Available at: https://goto.now/oDYrQ

[bibr32-00220574251318312] Government of Canada . (2022). The rise of ideologically motivated violent extremism in Canada. Government of Canada. Available at: https://goto.now/v4Md1

[bibr33-00220574251318312] Hafez M. Mullins C. (2015). The radicalization puzzle: A theoretical synthesis of empirical approaches to homegrown extremism. Studies in Conflict & Terrorism, 38(11), 958–975. 10.1080/1057610X.2015.1051375

[bibr34-00220574251318312] Harris-Hogan S. Barrelle K. Smith D. (2019). The role of schools and education in countering violent extremism (CVE): Applying lessons from Western countries to Australian CVE policy. Oxford Review of Education, 45(6), 731–748. 10.1080/03054985.2019.1612343

[bibr35-00220574251318312] Hassan G. Brouillette-Alarie S. Alava S. Frau-Meigs D. Lavoie L. Fetiu A. Varela W. Borokhovski E. Venkatesh V. Rousseau C. Sieckelinck S. (2018). Exposure to extremist online content could lead to violent radicalization: A systematic review of empirical evidence. International Journal of Developmental Science, 12(1-2), 71–88. 10.3233/DEV-170233

[bibr36-00220574251318312] Haye T. Patrick J. (2023). Far-right incursions on Canadian postsecondary campuses 2012- 2022: A qualitative content analysis. Canadian Journal for New Scholars in Education, 14(2), 86–95. https://journalhosting.ucalgary.ca/index.php/cjnse/article/view/77233

[bibr37-00220574251318312] Horgan J. (2008). From profiles to pathways and roots to routes: Perspectives from psychology on radicalization into terrorism. The Annals of the American Academy of Political and Social Science, 618(1), 80-94. 10.1177/0002716208317539

[bibr38-00220574251318312] Horgan J. Braddock K. (2010). Rehabilitating the terrorists? Challenges in assessing the effectiveness of de-radicalization programs. Terrorism and Political Violence, 22(2), 267–291. 10.1080/09546551003594748

[bibr39-00220574251318312] Hutchinson J. Amarasingam A. Scrivens R. Ballsun-Stanton B. (2021). Mobilizing extremism online: Comparing Australian and Canadian right-wing extremist groups on Facebook. Behavioral Sciences of Terrorism and Political Aggression, 15(2), 215–245. 10.1080/19434472.2021.1903064

[bibr40-00220574251318312] Jasko K. LaFree G. Kruglanski A. (2017). Quest for significance and violent extremism: The case of domestic radicalization. Political Psychology, 38(5), 815–831.

[bibr41-00220574251318312] Jungkunz S. (2019). Towards a measurement of extreme left-wing attitudes. German Politics, 28(1), 101–122. 10.1080/09644008.2018.1484906

[bibr42-00220574251318312] Kaldor M. (2012). New and old wars: Organized violence in a global era (3rd ed.). Polity Press.

[bibr43-00220574251318312] Karmon E. (2005). Coalitions between terrorist organizations: Revolutionaries, nationalists, and Islamists. Brill.

[bibr44-00220574251318312] Koehler D. (2014). The radical online: Individual radicalization processes and the role of the internet. Journal for Radicalization, 15(1), 116–134. https://journals.sfu.ca/jd/index.php/jd/article/view/8

[bibr45-00220574251318312] Korotayev A. Vaskin I. Tsirel S. (2019). Economic growth, education, and terrorism: A Re-analysis. Terrorism and Political Violence, 33(3), 572–595. 10.1080/09546553.2018.1559835

[bibr46-00220574251318312] Maiberg H. Kilp A. (2022). Extremism in a classroom: Topics discussed and Estonian teachers' experiences and self-reflective choices. Society Register, 6(1), 107–128. 10.14746/sr.2022.6.1.06

[bibr47-00220574251318312] Maraj-Guitard A. Mahmut D. Ghosh R. (2021). What role do French society and its education system play in promoting violent radicalization process? Journal for Deradicalization, 27(1), 238–283. https://journals.sfu.ca/jd/index.php/jd/article/view/469

[bibr48-00220574251318312] Maxwell J. A. (2013). Qualitative research design: An interactive approach (3rd ed.). Sage Publications.

[bibr49-00220574251318312] McCauley C. Moskalenko S. (2008). Mechanisms of political radicalization: Pathways toward terrorism. Terrorism and Political Violence, 20(3), 415–433. 10.1080/09546550802073367

[bibr50-00220574251318312] Miconi D. Geenen G. Frounfelker R. L. Levinsson A. Rousseau C. (2022). Meaning in life, future orientation and support for violent radicalization among Canadian college students during the COVID-19 pandemic. Frontiers in Psychiatry, 13(1), 765908. 10.3389/fpsyt.2022.765908 35222111 PMC8873191

[bibr51-00220574251318312] Miconi D. Santavicca T. Frounfelker R. L. Rousseau C. (2023). Preference for online social interactions and support for violent radicalization among college and university students. American Journal of Orthopsychiatry, 93(4), 350–363. 10.1037/ort0000681 37166896

[bibr52-00220574251318312] Moghadam A. (2009). The globalization of martyrdom: Al Qaeda, salafi jihad, and the diffusion of suicide attacks. Johns Hopkins University Press.

[bibr53-00220574251318312] Mucha W. (2017). Polarization, stigmatization, radicalization, counterterrorism and homeland security in France and Germany. Journal for Deradicalization, 10(1), 230–247. https://journals.sfu.ca/jd/index.php/jd/article/view/89

[bibr54-00220574251318312] Mudde C. (2007). Populist radical right parties in Europe. Cambridge University Press.

[bibr55-00220574251318312] Noddings N. (1988). An ethic of caring and its implications for instructional arrangements. American Journal of Education*,* 96 (2), 215–230. 10.1086/443894

[bibr56-00220574251318312] Parent R. Ellis J. O. (2016). The future of right-wing terrorism in Canada. Canadian Network for Research on Terrorism, Security and Society. TSAS Working Paper Series, 16-12.

[bibr57-00220574251318312] Perry B. Scrivens R. (2016). Uneasy alliances: A look at the right-wing extremist movement in Canada. Studies in *Conflict and terrorism*.

[bibr58-00220574251318312] Perry B. Scrivens R. (2019). Epilogue: The Trump effect on right-wing extremism in Canada. In Barbara P. Ryan S. (Eds.), Right-wing extremism in Canada (pp. 143–172). Palgrave Macmillan.

[bibr59-00220574251318312] Pickel G. Öztürk C. (2018). Islamophobia without Muslims? The “contact hypothesis” as an explanation for anti-Muslim attitudes – Eastern European societies in a comparative perspective. Journal of nationalism. Memory & Language Politics, 12(2), 162–191. 10.2478/jnmlp-2018-0009

[bibr60-00220574251318312] Preston K. (2023). Justifying contentious social and political claims using mundane language: An analysis of Canadian right-wing extremism. Current Sociology, 72(6), 1023–1048. 10.1177/00113921231182183 39493593 PMC11530338

[bibr61-00220574251318312] Public Safety Canada . (2017). 2017 public report on the terrorist threat to Canada. Public Safety Canada. Available at: https://www.publicsafety.gc.ca/cnt/rsrcs/pblctns/pblc-rprt-trrrst-thrt-cnd-2017/index-en.aspx

[bibr62-00220574251318312] Schmid A. P. (2011). The Routledge handbook of terrorism research. Routledge.

[bibr65-00220574251318312] Sjøen M. M. (2021). Let’s talk about terrorism. Journal of Peace Education, 18(3), 309–325. 10.1080/17400201.2021.1987869

[bibr66-00220574251318312] Sjøen M. M. (2023). The two faces of Janus: Educational pathways into and out of violent extremism in Norway. Journal of Peace Education, 20(2), 217–240. 10.1080/17400201.2023.2234836

[bibr67-00220574251318312] Sjøen M. M. (2024). Engaging with the elusiveness of violent extremism in Norwegian schools – the promise and potential of agonistic listening. British Journal of Educational Studies, 72(3), 321–340. 10.1080/00071005.2023.2251151

[bibr68-00220574251318312] Sjøen M. M. Mattsson C. (2019). Preventing radicalisation in Norwegian schools: How teachers respond to counter-radicalisation efforts. Critical Studies on Terrorism, 13(2), 218–236. 10.1080/17539153.2019.1693326

[bibr69-00220574251318312] Sklad M. Park E. (2017). Examining the potential role of education in the prevention of radicalization from the psychological perspective. Peace and Conflict: Journal of Peace Psychology, 23(4), 432–437. 10.1037/pac0000258

[bibr70-00220574251318312] Vallinkoski K. Benjamin S. (2023). From playground extremism to neo-nazi ideology -- Finnish educators' perceptions of violent radicalisation and extremism in educational institutions. Journal of Social Science Education, 22(1), 2. 10.11576/jsse-5288

[bibr71-00220574251318312] Vallinkoski K. Koirikivi P.-M. Malkki L. (2022). What is this ISIS all about?’ Addressing violent extremism with students: Finnish educators’ perspectives. European Educational Research Journal, 21(5), 778–800. 10.1177/14749041211010074

[bibr72-00220574251318312] Wang J. H. Moreau G. (2022). Police-reported hate crime in Canada, 2020. Statistics Canada. Available at: https://www150.statcan.gc.ca/n1/pub/85-002-x/2022001/article/00005-eng.htm

